# Programmable Potentials: Approximate N-body potentials from coarse-level logic

**DOI:** 10.1038/srep33415

**Published:** 2016-09-27

**Authors:** Gunjan S. Thakur, Ryan Mohr, Igor Mezić

**Affiliations:** 1 Harvard University, John A. Paulson School of Engineering and Applied Sciences, Cambridge, MA 02138, USA; 2 University of California Santa Barbara, Department of Mechanical Engineering, Santa Barbara, CA 93106, USA

## Abstract

This paper gives a systematic method for constructing an *N*-body potential, approximating the true potential, that accurately captures meso-scale behavior of the chemical or biological system using pairwise potentials coming from experimental data or ab initio methods. The meso-scale behavior is translated into logic rules for the dynamics. Each pairwise potential has an associated logic function that is constructed using the logic rules, a class of elementary logic functions, and AND, OR, and NOT gates. The effect of each logic function is to turn its associated potential on and off. The *N*-body potential is constructed as linear combination of the pairwise potentials, where the “coefficients” of the potentials are smoothed versions of the associated logic functions. These potentials allow a potentially low-dimensional description of complex processes while still accurately capturing the relevant physics at the meso-scale. We present the proposed formalism to construct coarse-grained potential models for three examples: an inhibitor molecular system, bond breaking in chemical reactions, and DNA transcription from biology. The method can potentially be used in reverse for design of molecular processes by specifying properties of molecules that can carry them out.

Multi-body interactions are ubiquitous in nature and happen at all scales from atomic (quantum description) to molecular (classical approach) to macro scales. A systematic analysis these interactions may unfold the fundamental principles governing a given system. For example, understanding the biophysics of protein folding gives insight into disease pathologies^1^. This understanding can be leveraged to develop new vaccines and drug therapies. Engineering these new products requires accurate and computationally tractable models.

Systems having multibody interactions, in fundamental physics, are often formulated as a “*N*-body potential” problem. In order to fully understand these systems a large number of experiments are needed. Conducting experiments may be expensive and at times even impossible. Another approach is to analyze the *N*-body potential governing the system dynamics. However, at the quantum level, it may be difficult to determine these potentials from first principles due to the complexity of the system. The computational complexity for ab initio methods can scale exponentially in the number of electrons, limiting the practical size of the system to a few thousand atoms^2^,^3^,^4^. Even if the detailed potential is determined, it may not be immediately useful. Such is the case when the properties or behaviors of interest are at a coarser level than that of the detailed potential and simulating the detailed dynamics is too expensive. Very coarse approaches such as those of master equation^5^ lack predictability on molecular spatial and time scales due to the assumptions with which they are derived. A potential that models the system is required if one is to make predictions about the system.

It is profitable to restrict one’s efforts to considering approximate potentials that respect known behavior. Such coarse-level descriptions may be determined from experimental observation and may correspond to trajectories in some transformed (reaction) coordinate system. For example, consider a signal transduction mechanism^6^,^7^,^8^,^9^,^10^, hierarchical self-assembly^11^,^12^,^13^,^14^,^15^,^16^,^17^,^18^,^19^,^20^, Kinesin motor protein translocation on a microtubule^21^,^22^, or hydrogen combustion H_2_/O_2_^23^,^24^. These systems transition from one stable configuration to another on the occurrence of some trigger event which may comprise of an external stimulus or the system reaching a special configuration. An external stimulus could be an input of energy that initiates hydrogen combustion, leading to a larger release of energy by the reaction itself. A special configuration could be a signaling molecule binding to an active receptor site. These stable configurations can be considered as fixed points in a transformed (reaction) coordinate system. The fixed points, the events, and their associated transitions are the coarse-level descriptions that are to be captured in the approximate *N*-body potential. However, it is still a challenge to construct a *N*-body Hamiltonian potential in a systematic manner that encodes the known coarse-level behaviors into a mathematical formulation and successfully predicts intermediate-scale transition events.

This article introduces a method of encoding coarse-level dynamical behavior into logic functions that are used to “stitch” together pairwise interaction potentials into an *N*-body potential. In this method, the practitioner uses experimentally observed coarse-level behavior to derive logic tables that capture various rules of interaction in the system. The qualitative logic tables are turned into a collection of quantitative logic functions associated with pairwise interaction potentials. The logic functions are then turned into smooth encoding functions via a replacement procedure which in turn are used to modify the pairwise potentials. The effect of an encoding function multiplying a pairwise potential is to smoothly turn the potential on or off when a precise set of conditions are met. The combination of the modified potentials gives an *N*-body potential that approximates the true potential governing the system.

The method generates a potential that respects what is currently known about the system; it is not claimed that this method results in the unique potential governing the real system. The method does this by leveraging the existing experimental data and the coarse-level behavior that can be derived from it. If more experimental data becomes available, the same procedure can be used to generate a new potential that better models the system. This is equivalent to a refinement of the logic functions and ultimately a refinement of the generated potential. The resulting potential can have a much smaller dimension than the true potential and still accurately capture the relevant physics.

This article begins with a motivating example which is used as an impetus for our modeling framework. In the Methodology section, we define the major components of the framework — logic functions, permissible logical operations, and the translation to the associated encoding functions — and specify how they combine with the pairwise potentials to define the approximate potential. The procedure is depicted in Fig. 1.

The procedure is applied to three examples of increasing complexity to showcase the modeling framework. The first is a simple model of an inhibitor molecule mechanism. It shows how one would go from known coarse-level behavior to an approximate global potential that captures that behavior by explicitly constructing the logic tables, the associated logic functions, and the smooth encoding functions. The inhibitor molecule mechanism has more complicated logic than the motivating example and more effectively demonstrates the modeling procedure.

The second example shows how to model a simple, kinetically controlled, bond breaking chemical reaction using this framework. It shows that bond breaking events, and more generally chemical reactions requiring activation energy, can be naturally modeled in the framework. The general procedure for modeling a bond breaking event and how to account for the activation energy is shown. Furthermore, the derived potential is used with LAMMPS^25^ to numerically simulate the chemical reaction. By changing the relative dissociation energies, the reaction can be biased in a particular direction.

As opposed to our method, many force fields have trouble capturing bond breaking events^26^. An exception is the ReaxFF potential^2^ that was developed to model reactions of hydrocarbons. The derivation of ReaxFF is based on using interatomic distances to compute the bond order between two atoms and then using the bond order to obtain the bond energy. Corrections to the bond order are dependent on the valency and the deviation of the uncorrected bond order of an atom with its valency. Corrections to the bond energy, in the form of energy penalties (e.g. for over-/under-coordination) are added to get the system energy. This is contrasted with our method where bond weakening and breaking is due to the encoding function which is derived from coarse-level observed behavior.

The final example is a simple model of DNA transcription. It is shown that after the binding of RNA polymerase to the promoter region we can sequentially add the complementary base nucleotides to the DNA strand that is to be transcribed. DNA transcription is a complex process involving the interaction of many different molecules^27^,^28^. This example shows that we can model such a complex process with a relatively low-dimensional potential that captures the observed mesoscale behavior. To the authors’ knowledge there is no other other current potential accomplishing this task.

## Motivating Example

**Figure 2:**
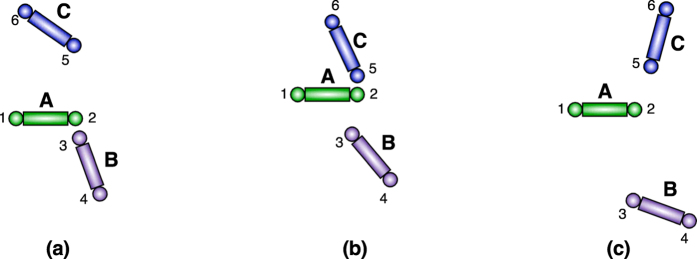
Simple signaling molecule mechanism, 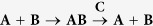, modeled by three rods. When **C** is not present, **A** and **B** form a complex. When **C** is present, **A** and **B** dissociate and diffuse apart. Molecule **C** is free to diffuse away from molecule **A**. This behavior is captured with the following rules. When atom 5 is far from atom 2, the potential between atoms 2 and 3 is on. When atom 5 is close to atom 2, the potential between atoms 2 and 3 is turned off allowing molecules **A** and **B** dissociate and diffuse apart. Atom 5 can diffuse away from atom 2.

There are a number of examples in biology where chemical reactions occurring within a cell are initiated by some signal or stimulus, followed by an ordered sequence of biochemical reactions. Often the term signal transduction is used to refer to such processes. One such example is the epidermal growth factor (EGF) signaling^9^,^10^. Motivated by this example, we construct a hypothetical system to demonstrate how the proposed formulation can be used to construct a Hamiltonian potential for it. Assume a system of three species, **A**, **B** and **C**, has an evolution dictated by the chemical equation 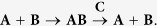 The sequence in which these reactions happen define logical “interaction rules” used to design the potential. Specifically, these rules are (1) when molecules **A** and **B** are close, and **C** is far, then **A** and **B** bond; and (2) If **C** approaches the **AB** complex, then **A** and **B** dissociate. This mechanism is visualized in Fig. 2.

Each of the species in this system can be modeled as a rod having two sites of interaction at the end points; atoms {1, 2} on **A**, atoms {3, 4} on **B**, and atoms {5, 6} on **C** (Fig. 2). Let us write the force field energy for this system. In general, it is composed of the bonded energies formed from the stretch, bending, and torsional terms, the non-bonded van der Waals and electrostatic terms, and the coupling terms^26^. We can split the potential as


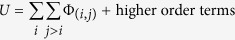


where Φ_(*i*,*j*)_ denotes a pair-wise interaction potential between two atoms *i* and *j*. These 2-atom terms encompass the stretching, torsional, van der Waals, and electrostatic terms and the higher order terms include the bending energies and all the *k*-atom (*k* ≥ 3) coupling terms.

For this system, the bonded energy terms are composed of the stretch energies between the atom-atom pairs (1, 2), (3, 4) and (5, 6), which we can group into a term *U*_*b*_. Assume that the only non-negligible non-bonded energy terms are the two van der Waals interactions between atoms 2 and 3 and atoms 2 and 5, and the coupling term between atoms 2, 3, and 5. Denoting these three terms by Φ_(2,3)_, Φ_(2,5)_, and Φ_(2,3,5)_, respectively, we get the force field energy of the system as


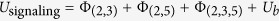


The inclusion of the 3-atom potential Φ_(2,3,5)_ is required in order to capture the transition of the pair (2, 3) being in a stable (bounded) configuration when atom 5 is not present to being in an unstable (free) configuration in the presence of the signaling atom 5.

While in general it may be hard to get the correct forms for the coupling term and other higher-order terms in the expansion, and thus the full potential, we know from the above observations that the effect of the potentials Φ_(2,5)_ and Φ_(2,3,5)_ is to basically to turn off Φ_(2,3)_ when 5 is close to 2. Rewriting the potential as


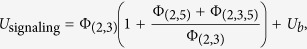


this means that term in parentheses is approximately 1 whenever atoms 2 and 5 are far and approximately 0 whenever atoms 2 and 5 are close. Instead of attempting to find the exactly functional forms of Φ_(2,5)_ and Φ_(2,3,5)_, we approximate the potential as


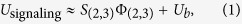


where *S*_(2,3)_ is an encoding function that acts as a switch turning Φ_(2,3)_ on and off. In this example, the encoding function is only function the distance between atoms 2 and 5. The encoding function takes values between 0 and 1, it is approximately 0 when atoms 2 and 5 are close, and approximately 1 when atoms 2 and 5 are far; thus it encodes the logic of the coarse-level observed behavior of the system. It is an approximation of the other terms:


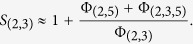


In the rest of the article, we make this approximation idea (Eq. (1)) precise and derive approximate *N*-body potentials from simple pairwise interactions that respect observed coarse-level behavior. We give a systematic procedure to construct the encoding functions which allows us to handle systems with more complex interaction rules. We will demonstrate the procedure with three examples. We also use molecular dynamics simulations using the derived potentials that show we can accurately capture the relevant physics.

There are a few items to keep in mind as motivation for the abstract concepts to follow. The basic building blocks for the *N*-body potential are pairwise interaction potentials (denoted by Φ_(2,3)_ and Φ_(2,5)_ for the above example). The explicit form of these potentials can be inferred from the experimental data or ab initio calculations. We approximate the effect of the un-modeled potentials by modifying the relevant pairwise potentials with an encoding function. The encoding function only turns the corresponding potential on and off. The functional form of the potential does not change; it is only scaled between 0 and 1. The logic contained in the encoding functions is obtained from experimental observations or ab initio simulations and the logic only depends on pairwise distances between particles, except the pairwise distance used in the associated potential function; e.g. the logic corresponding to Φ_(2,3)_ cannot depend on the distance between atoms 2 and 3.

## Methodology

It is assumed that there are *M* interacting entities in a domain 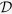, where 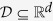, for *d* = 1, 2, or 3. Each entity is modeled by a finite number of particles with constraint forces between the particles; the totality of these particles over all the entities are labeled from 1 to *N*. This allows us to treat point particles as well as rigid and and non-rigid bodies. The configuration space is 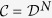. A particular system configuration, 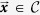, takes the form 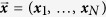, where 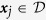 describes the position of particle *j* in the domain 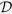.

The dynamics of the system is driven by a potential gradient and external forces. Specifically, the functional form of the dynamics is


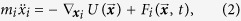


where *m*_*i*_ is the mass of atom *i*; 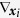 is the gradient operator in the configuration space 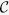 with respect to the the position ***x***_*i*_ of atom *i*; 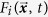 collects the external forces on particle *i* such as external electric or magnetic fields as well as stochastic effects or boundary constraints; and the approximate potential is defined as


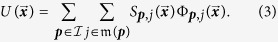


The notation in this equation is as follows.

### 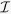 , set of interacting pairs of atoms


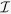
 defines which pairs of atoms interact. For example, if ***p*** = (2, 3) is in 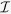, then there exists a pairwise potential between atoms 2 and 3. Since not every pair of atoms in the system has to interact, 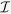 can be a strict subset of {1, …, *N*} × {1, …, *N*}.

### 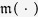 , multiplicity function

The multiplicity function 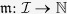 determines how many potentials that atom pair ***p*** = (*p*_1_, *p*_2_) interacts through. Often 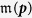 will be 1 for every atom pair ***p***. However, non-unit values become important when a pair 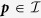 interacts through multiple different potentials, each with its own encoding function. For example, a non-unit multiplicity is useful when modeling bond-breaking chemical reactions. Initially, two atoms interact through their bond potential; when this bond is broken, another potential is required to model the electron-electron repulsion between the atoms.

### Φ_***p***,*j*,_ pairwise interaction potential


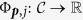
 is the *j*^*th*^ interaction potential for the pair of atoms 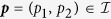. The index *j* is runs from 1 to 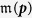. For 
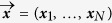
, it takes the form


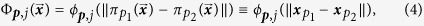


where for every *i* ∈ {1, …, *N*}, the coordinate map 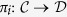 extracts the position ***x***_*i*_ of atom *i* from the configuration vector 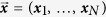; the norm 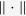 denotes the normal Euclidean norm; and 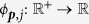 is the *j*^*th*^ 1D pairwise interaction potential through which the pair ***p*** interacts. This potential could be, for example, a Lennard-Jones or Morse potential; it can also be different for different interaction pairs. The form given for the potential shows that it is only a function of the distance between 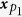 and 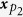. When 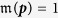, we drop the *j* index from Φ_***p***,*j*_ and write it as Φ_***p***_.

### *S*_***p***,*j*_, encoding function


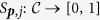
 is the encoding function associated with the potential Φ_***p***,*j*_. It encodes the coarse-level interaction rules and it is a function of pairwise distances between particles, except for the particle pair ***p*** to which it corresponds. That is, for the interaction pair 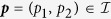, the encoding function *S*_***p***,*j*_ is *not* a function of the distance 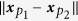. The effect of *S*_***p***,*j*_ is to smoothly turn its associated potential function on and off based on the configuration of the system. Since the encoding functions and potentials are functions of relative distances only, Equation (2) defines a Hamiltonian system^29^ when we neglect the forces 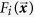. When 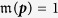, we drop the *j* index from *S*_***p***,*j*_ and write it as *S*_***p***_.

A majority of the rest of the paper develops the encoding functions and their properties and shows how one would go from coarse-level interaction rules to encoding functions using a few examples. It is assumed that the coarse-level, interaction rules and the interaction potentials 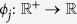 are known. These come from analyzing experimental data or ab initio simulations and are thus application specific and beyond the scope of this article. Ultimately, the encoding function *S*_***p***,*j*_ will be a smoothed version — which is made precise later — of a logic function 
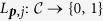
, which assigns 0 or 1 to each configuration 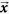. The logic function *L*_***p***,*j*_ will be the constructed from a finite number of logical operations applied to elementary logic functions from a Boolean algebra. More precisely, the logic function will be an element of the Boolean sub-algebra generated by elementary logic functions. Thus to define the logic functions, it is required to know the specific definitions of the logical operators AND, OR, and NOT (symbolically denoted, ∧, ∨, ¬) and what Boolean functions are used as the elementary logic functions.

A function 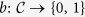, which assigns either 0 or 1 to each configuration vector 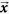 is called a Boolean function on 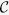 and the set of such all such functions on 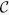 is denoted as 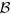. It is easy to see that the functions that are identically 1 and 0 on 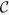 are Boolean functions. On 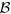, define for all 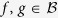 the two binary logical operations AND (∧) and OR (∨) and the unary logical operation NOT (¬) by


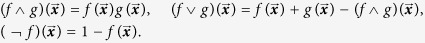


These three logical operations will be applied to specific elements of the set of all Boolean functions 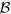 on 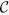 to generate a Boolean sub-algebra. The logic functions *L*_***p***,*j*_ will be elements of this sub-algebra.

Proximity functions are used to define the elementary logic functions. A proximity function 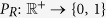 has the form *P*_*R*_(*r*) = *χ*_[0,*R*)_(*r*), for some *R* satisfying 0 ≤ *R* ≤ ∞. The function 
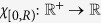
 is the indicator function for the semi-open interval [0, *R*) which takes the value 1 if the argument satisfies 0 ≤ *r* < *R* and 0 otherwise. Note that the functions that are identically 1 or 0 are proximity functions. The *elementary logic functions* are defined as compositions of a proximity function with the coordinate functions *π*_*i*_ from above. Specifically, the elementary logic function 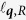 for atom pair 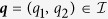 and parameter 0 ≤ *R* ≤ ∞ has the form


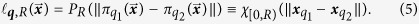


This function is 1 when 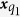 and 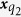 are closer than distance *R* and 0 when not.

A logic function *L*_***p***,*j*_ is generated by applying finitely many of the logical operations ∧, ∨, and ¬ to the elementary logic functions (5) for any finite set of ***q***’s, none of which are equal to ***p*** — that is, 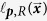 cannot be part of the definition of *L*_***p***,*j*_. Each logic function *L*_***p***,*j*_ is continuous almost everywhere in 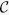 since each elementary logic function is constant almost everywhere. This follows since it is composed from pairwise products and sums of elementary logic function, which themselves are continuous almost everywhere.

**Figure 3:**
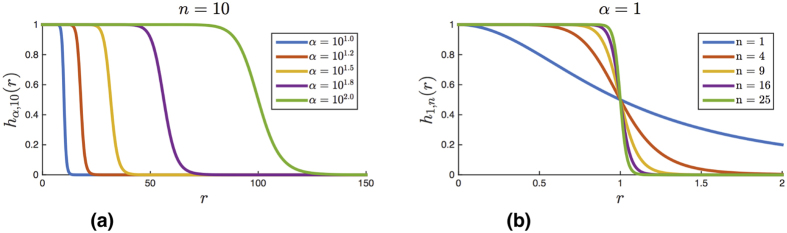
Behavior of the *h*_*α,n*_ function fromEquation (6). (**a**) *α* controls the transition point. (**b**) *n* controls the sharpness of the transition.

Once the logic function is specified, it must be translated into a smooth encoding function. Ideally, this would be accomplished via a convolution in the (*dN*-dimensional) configuration space with a smooth, nonnegative summability kernel (see Katznelson^30^ for a definition). Analytically, this is intractable, and computationally, this is very expensive. Instead, we individually smooth each of the 1D elementary logic functions 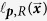 in the expression for 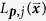. This is done by replacing the proximity function of 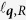 with a smoothed version. Again, this could be done via the convolution (now 1-dimenional) of each proximity function with a smooth, 1D summability kernel or, alternatively, by the replacement of each indicator function with a specific functional form. We choose the latter approach and replace each proximity function *χ*_[0,*R*)_(*r*) with a function of the form


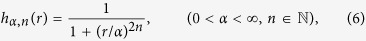


and we define *h*_0,*n*_(*r*) = 0 and *h*_∞,*n*_(*r*) = 1. For example, if the logic function has the expression


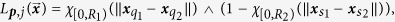


then the corresponding encoding function would be


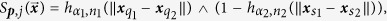


for some choices of parameters *α*_1_, *α*_2_, *n*_1_, and *n*_2_. The parameters *α* and *n* control how well *h*_*α*,*n*_ approximates a proximity function (see Fig. 3). In particular, *h*_*α*,*n*_(0) = 1 for any 0 < *α* < ∞ and positive *n*. Furthermore, 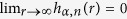 and it is strictly monotonically decreasing. On the other hand, for a fixed 0 < *α* < ∞, the transition from 1 to 0 becomes sharper as *n* increases (Fig. 3(b)). To match a specific indicator function *χ*_[0,*R*)_, choose *α* = *R*. With this choice of *α*, the function satisfies *h*_*R*,*n*_(*R*) = 1/2 for all *n* ≥ 1;


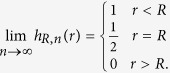


## Examples

To show the entire process, starting from coarse, interaction rules and recovering the encoding function, we apply the method to three examples in increasing order of complexity. The first example is a model for an inhibitor molecule system and is used to exhibit the core methodology of the modeling framework. This system can be considered as an extension of the signaling molecule example above (Fig. 2). The second example is a model for a simple bond breaking chemical reaction and makes use of the multiplicity function 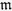 from the framework. It is shown that the bond dissociation energy is accurately captured in this framework. Numerical simulations show that (i) the system exhibits the same coarse-level behavior that was used to derive the potential and (ii) that biased chemical reactions are easily handled. The final example is a simple model for DNA transcription and is the most complicated of the three. This example shows that the logic, and hence potential, of real systems can be captured in the modeling framework in a straight-forward manner.

### Simple inhibitor molecule mechanism

**Figure 4:**
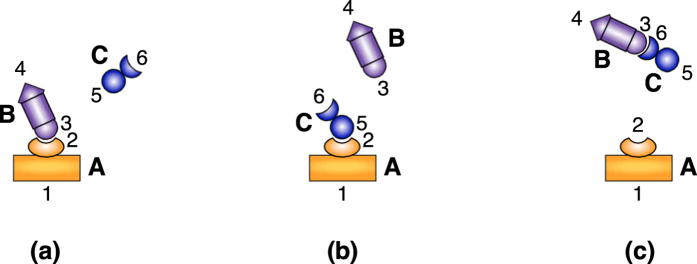
Inhibitor molecule example. When the inhibitor molecule, **C**, is not present (panel **(a)**), a bond between receptor **A**’s site 2 and site 3 on the active molecule **B** can form. When the inhibitor molecule is present, it can either take up the receptor site through a (2, 5) bond (panel **(b)**) or bind to site 3 on **B** with site 6 (panel (**c**)). Either of these cases prevents to active molecule **B** from binding with its receptor site on **A**.

This example can be thought of as a simple model for the action of an inhibitor molecule in a plane. Consider the three interacting molecules in Fig. 4. The configuration space for this example is 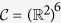, where 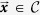 is written as 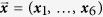. The set of interacting atom pairs is 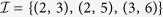. For this example, the multiplicity function 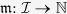 is identically 1. Thus we have the pairwise potentials Φ_(2,3)_, Φ_(2,5)_, and Φ_(3,6)_. It is assumed that these potentials are formed using a Morse potential (see (12)). Molecule **C** is an inhibitor molecule and prevents the formation of the **AB** complex. Without **C**, we have **A** + **B** → **AB**. With **C** present, the there are two possibilities: (i) 
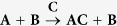
 or (ii) 
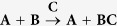
.

This behavior is captured in the logic functions *L*_(2,3)_, *L*_(2,5)_ and *L*_(3,6)_. The logic function *L*_(2,3)_ is 0, i.e., the potential Φ_(2,3)_ is turned off, when either atom 5 is close to atom 2 or when atom 6 is close to atom 3. This is different from the motivational example which only turned off the potential if 2 and 5 were close and the bonds between 2 and 5 or 3 and 6 never formed. Additionally, if *AC* has formed (atoms 2 and 5 close), then *BC* cannot form, i.e., *L*_(3,6)_ = 0 and Φ_(3,6)_ is off. Similarly, *BC* has formed (atoms 3 and 6 close), then *AC* cannot form. Table 1 captures this logic. As a general rule when determining the logic, the default state for all the potentials should be set to “on” except when encoding a specific mechanism. In this example, this corresponds to the first row of Table 1 which says that the values of all the logic functions are 1 when all of the atoms are far apart. This means that the associated potentials are turned on. This is exactly the behavior we want since the inhibitor mechanism is inherently a short range phenomena and thus we do not want the mechanism to be active when all the particles are far apart. However, since the atoms are all far apart the long-range behavior of the potential is dominant. For a Lennard-Jones or a Morse potential, the means there is a weak attraction force between the pairs of atoms.

We need to specify what is meant by “close”. We assume that “close” is in this case is determined by experiments to mean being within the distances *R*_(2,5)_ and *R*_(3,6)_, respectively. Thus, atoms 2 and 5 are close when the elementary logic function 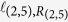 evaluates to 1 and not close when it evaluates to 0. Using the table, 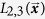 corresponding to the interaction potential 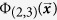 can be written as Equation (7).


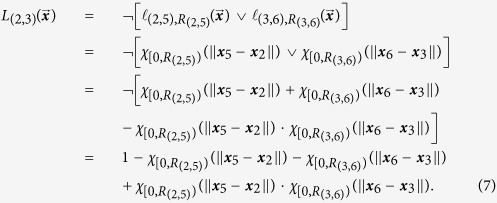


The other logic functions are


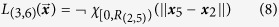



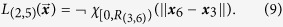


To turn the logic functions into an encoding function, replace each of the proximity functions, 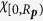, in (7–9) with their smooth versions, 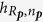 (Eq. (6)). The encoding function *S*_(2,3)_ corresponding to *L*_(2,3)_ is


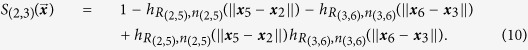


The approximate potential for this system is


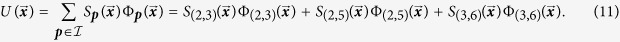


The original configuration space was 12-dimensional. However, (11) is 8-dimensional since it only depends on four atoms (four unique atoms making pairs in 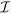). Thus we were able to reduce the dimension of the configuration space and still capture the relevant physics.

**Figure 5:**
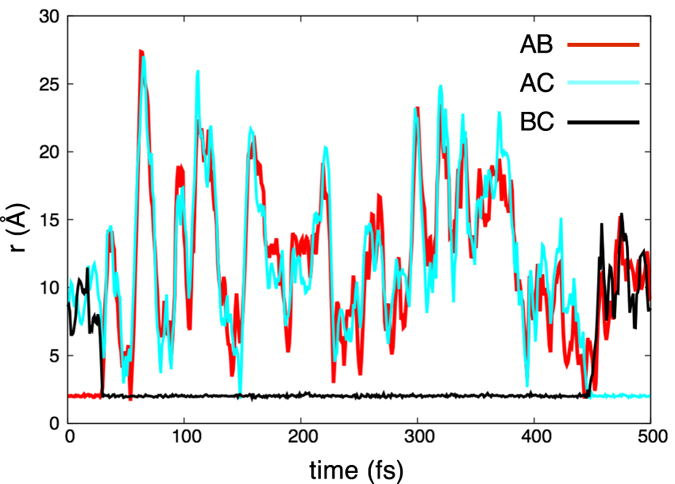
One realization of the inhibitor molecule system (11) simulated in LAMMPS. Initially, the **AB** complex is formed. Around 30 femtoseconds (fs) **C** comes close enough, turns off the **AB** bond and **BC** forms and can diffuse away from **A**. This remains the case until around 450 fs, when **A** approaches **BC**, the **BC** bond turns off and the **AC** bond turns on.

Here, we are only interested in demonstrating the methodology qualitatively so we make the approximation that derivative of each encoding function is 0 almost everywhere (this would be the case if the logic functions were used in place of the encoding functions in (11). With this approximation the force only consists of terms of the form 
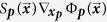
. One realization of the inhibitor molecule system (11) simulated in LAMMPS^25^ with this approximation is shown in Fig. 5. The potentials Φ_***p***_ are formed from Morse potentials


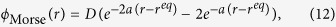


where *D* is the dissociation energy, *r*^*eq*^ is the equilibrium distance of the bond, and *a* is a parameter. Simulations are performed by solving the Langevin equations at constant temperature (i.e. NVE ensemble). The parameters used in the computations are given in Supplementary Table I. Initially, the **AB** complex is formed. Around 30 femtoseconds **C** comes close enough, turns off the **AB** bond and **BC** forms and can diffuse away from **A**. This remains the case until around 450 fs, when **A** approaches **BC**, the **BC** bond turns off and the **AC** bond turns on.

Supplementary Movie 1 shows a simulation of the inhibitor molecule mechanism.

### Modeling a bond breaking chemical reaction

We model a reversible, bond breaking, chemical reaction. In particular, we will model the reaction


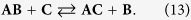


Modeling such chemical reactions is difficult with traditional force field methods since they cannot describe changes in the electronic structure and, thus, are unable to describe bond-breaking, bond-forming, charge transfer, etc., of the system undergoing a reaction^4^,^31^. Rather than solving the quantum mechanical equations, we take a coarse-level approach and approximate the bond breaking mechanism with logic functions.

**Figure 6:**
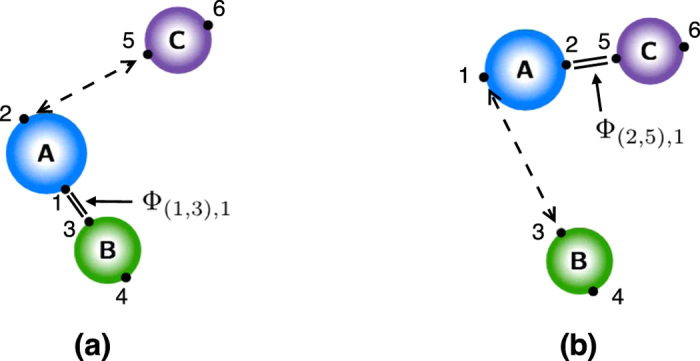
Diagram for chemical reaction example. Φ_(1,3),1_ and Φ_(2,5),1_ represent the stable bonds **AB** and **AC**, respectively. The dashed lines represent the repulsion forces induced by the encoding functions. (**a**) the repulsion between **A** and **C** is due to the partial derivatives of 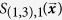 with respect to both ***x***_2_ and ***x***_5_. (**b**) the repulsion between **A** and **B** is due to the partial derivatives of 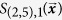 with respect to both ***x***_1_ and ***x***_3_. See Supplementary Information Sec. III.A for a discussion of the repulsion force induced by the smooth encoding functions.

This example makes use of the multiplicity function 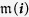 in (2) in order to model the electron-electron repulsion during the transition state. It also shows that the use of a smooth encoding function accurately accounts for the bond dissociation energy. Let 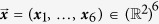 be the configuration vector for this system (see Fig. 6).

Table 2 lists all the potentials involved in modeling the system. The potential Φ_(1,3),1_ is the potential energy of the bond between **A** and **B** when they form the stable molecule **AB**, for example a Lennard-Jones or Morse potential. Similarly, Φ_(2,5),1_ is the bond potential energy between **A** and **C** when they form the stable molecule **AC**. They have the associated logic functions *L*_(1,3),1_ and *L*_(2,5),1_, respectively. The potentials Φ_(1,3),2_ and Φ_(2,5),2_ are used to model electron-electron repulsion during the transition state when the reactant bonds have broken and the product bonds have not yet formed. The secondary potentials are usually taken to be the repulsive part of the associated bond potential.

Table 3 lists the logic rules for this system. Consider the forward reaction **AB** + **C** → **AC** + **B**. We model the bond breaking mechanism by turning off the stable bond, Φ_(1,3),1_, when **C** gets “close enough” to **A**. When **C** moves within the distance 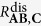 to **A**, the **AB** bond (Φ_(1,3),1_) turns off. Similarly, for the backwards reaction, the **AC** bond (Φ_(2,5),1_) turns off when **B** gets within a distance 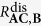 of **A**. The logic functions are logical NOT’s of the ***x***_2_–***x***_5_ and ***x***_1_–***x***_3_ proximity functions:


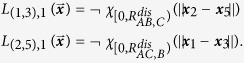


We assume that 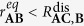 and 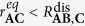. With this assumption, the **AC** bond turns off before **B** reaches its equilibrium bond length with **A**.

The use of the smooth encoding function in the potential (as opposed to the logic function) allows the transfer of the correct amount of energy from **C** to **AB** in order to break the bond; **C** must transfer an amount of energy equivalent to the bond dissociation energy *D*_**AB**_ of the **AB** bond in order to turn off the Φ_(1,3),1_ potential. We refer the reader to Sec. III.A in the Supplementary Information for the derivation.

Consider the situation occurring directly after a successful collision of **C** with **AB**. In this case, **A** and **B** are close to their equilibrium distance (
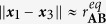
) and **A** and **C** are closer than the **AB**-bond dissociation distance (
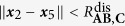
). In this state, the bonds are weak and neither **AB** nor **AC** is stable; the system is at its transition state. In this transition state the forces experienced by the molecules due to the bond potentials Φ_(2,3),1_ and Φ_(2,5),1_ are small since the encoding functions and their partial derivatives are small, and thus the bond potentials are approximately “off”. The dynamics are predominantly dominated by noise and the residual momentum of the molecules.

In this transition state, the electron-electron repulsion should be directly accounted for via a short-range repulsion potential between the molecules; usually this is repulsive part of the associated bond potential. The logic functions are defined such that these repulsion forces are only “on” when the system is in its transition state. This is easily accomplished. Denote the short-range repulsion potential between **A** and **C** by Φ_(2,5),2_. This force is defined such that 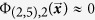, for 
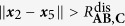
. This force is turned on when 
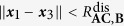
. The logic function for the **A**-**C** repulsion is


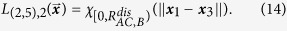


Similarly, repulsions between **A**-**B** and **B**-**C** can be defined with logic functions similar to the above one.

**Figure 7:**
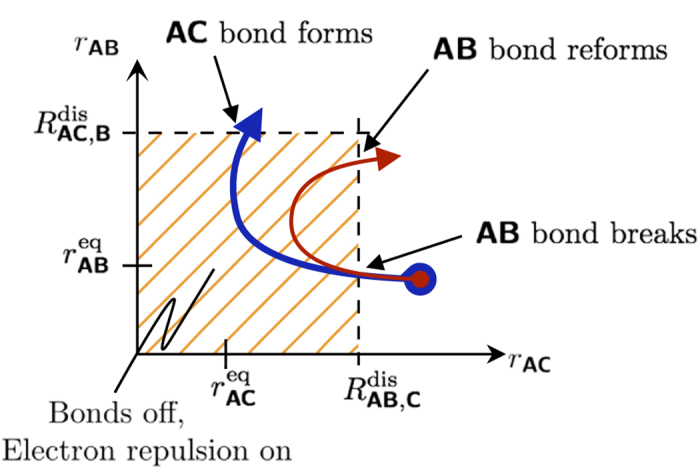
The two most probable outcomes of a successful AB + C event. Both trajectories start at the same configuration the difference is that **C** has a greater momentum for the blue (thicker) curved arrow. For both the red and blue trajectories, **C** approaches **A**. The **AB** bond breaks when 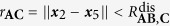. Depending on the momentum and the relative strength of the repulsion terms, either **AB** reforms or **AC** forms.

There are three possible outcomes for when the system exits its transition state: (i) either **AC** forms a stable molecule, (ii) **AB** reforms, or (iii) no bonds are formed and all the molecules are free molecules. This depends on the equilibrium distances of the bonds, the dissociation distances, the incoming momentum of **C**, and the repulsion forces. Figure 7 shows the two most probable outcomes for a single **AB** + **C** event.

For our simulations, the **AB** and **AC** bonds (Φ_(1,3),1_ and Φ_(2,5),1_, respectively) are given by Morse potentials, (12). In simulations, only the short-range **A**-**B** and **A**-**C** electron-electron repulsions are modeled and are only active during the transition state. The form of these for these repulsions are chosen as the repulsive part of a Morse potential with the same parameters as the full potentials used for the **AB** and **AC** bonds. The logic function for the **A**-**C** repulsion potential, Φ_(2,5),2_, is given by (14) with obvious modifications for Φ_(1,3),2_. The associated encoding functions are given by the normal replacement procedure. The full potential used during the numerical experiments is given in (15).


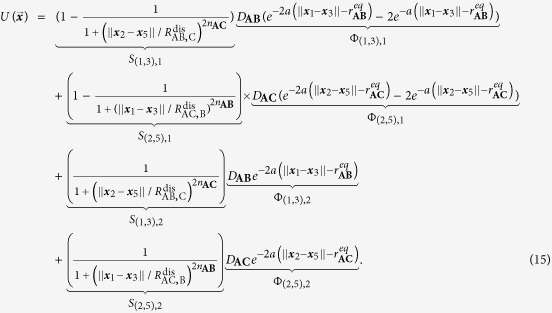


**Figure 8:**
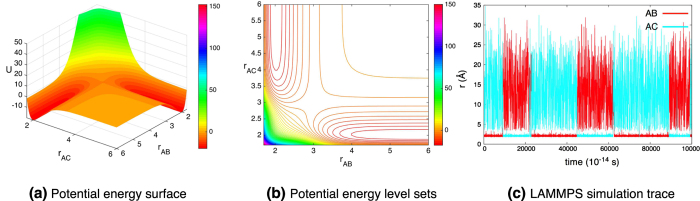
Simulation of an unbiased (1:1 well-depth), bond breaking chemical reaction, (13). (**a**) The potential energy (15) for the system. The parameters are given under simulation 1 in Supplementary Table II. (**b**) The level set plot of the potential energy. (**c**) A typical trajectory of the simulation. The cyan trace denotes the distance between molecules **A** and **C** (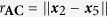), whereas the red trace corresponds to the distance between molecules **A** and **B** (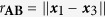). Initially, **A** and **C** are near their equilibrium length (2) and **B** is far from **A**. We see a successful **AC** + **B** → **AB** + **C** event happening very soon (red trace is close to the equilibrium distance, then becomes large; cyan trace is large, then becomes small).

The force derived from (15) is used in LAMMPS^25^ to simulate the system for an unbiased and a biased potential (parameters in Supplementary Information Table II). The parameters of the first simulation are chosen so that the **AB** and **AC** are symmetric (*D*_**AC**_/*D*_**AB**_ = 1). In this case, the chemical reaction is unbiased and if averaged over all realizations of the noise, it is expected that the amount of time **AB** is formed is equal to the amount of time **AC** is formed. Figure 8 shows the potential energy for this simulation (Fig. 8(a)), the corresponding level sets (Fig. 8(b)), and a typical realization of the simulation (Fig. 8(c)). In the energy surface plot and the level set plot, the symmetry of the potential is evident. The realization shown in Fig. 8(c) starts with **AB** near its equilibrium length (2 Å) with **C** far from **A**. The realization shows the approximately equal times that **AB** and **AC** are formed. The deviation is due to this being a particular realization rather than an average over an ensemble of realizations and the finite nature of the simulation.

**Figure 9:**
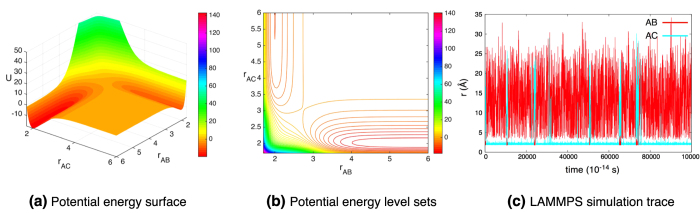
Simulation of biased (2:1 well-depth), bond breaking chemical reaction, (13). (**a**) The potential energy (15) for the system. The parameters are given under simulation 2 in Supplementary Table II. (**b**) The level set plot of the potential energy. (**c**) A typical trajectory of the simulation. The cyan trace denotes the distance between molecules **A** and **C** (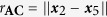), whereas the red trace corresponds to the distance between molecules **A** and **B** (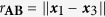). Initially, **A** and **B** are near their equilibrium distance (2) and **C** is far from **A**. We see a successful **AB** + **C** → **AC** + **B** event happening very soon (red trace is close to the equilibrium distance, then becomes large; cyan trace is large, then becomes small). The trace exhibits the bias towards a stable **AC** bond, since the cyan trace is close to equilibrium longer than the red trace.

The parameters of the second simulation are chosen so that the reaction is biased in favor of **AC**. With the chosen parameters (*D*_**AC**_/*D*_**AB**_ = 2), the **AC** bond is twice as stable as **AB**. Figure 9 shows the potential energy for this simulation (Fig. 9(a)), its corresponding level sets (Fig. 9(b)), and a realization of the simulation (Fig. 9(c)). In the energy surface plot and the level set plot, the asymmetry of the potential is evident. The realization shown in Fig. 9(c) starts with **AB** near its equilibrium length (2 Å) with **C** far from **A**. In this particular realization **AC** forms very quickly. Figure 9(c) shows the bias towards the more stable **AC**. The system spends most of its time with a stable **AC** molecule with a relatively small amount of time with a stable **AB** molecule. Thus, biased reactions can be captured in the framework. A movie of a part of the unbiased reaction simulation can be found in Supplementary Movie 2.

### DNA transcription model

**Figure 10:**
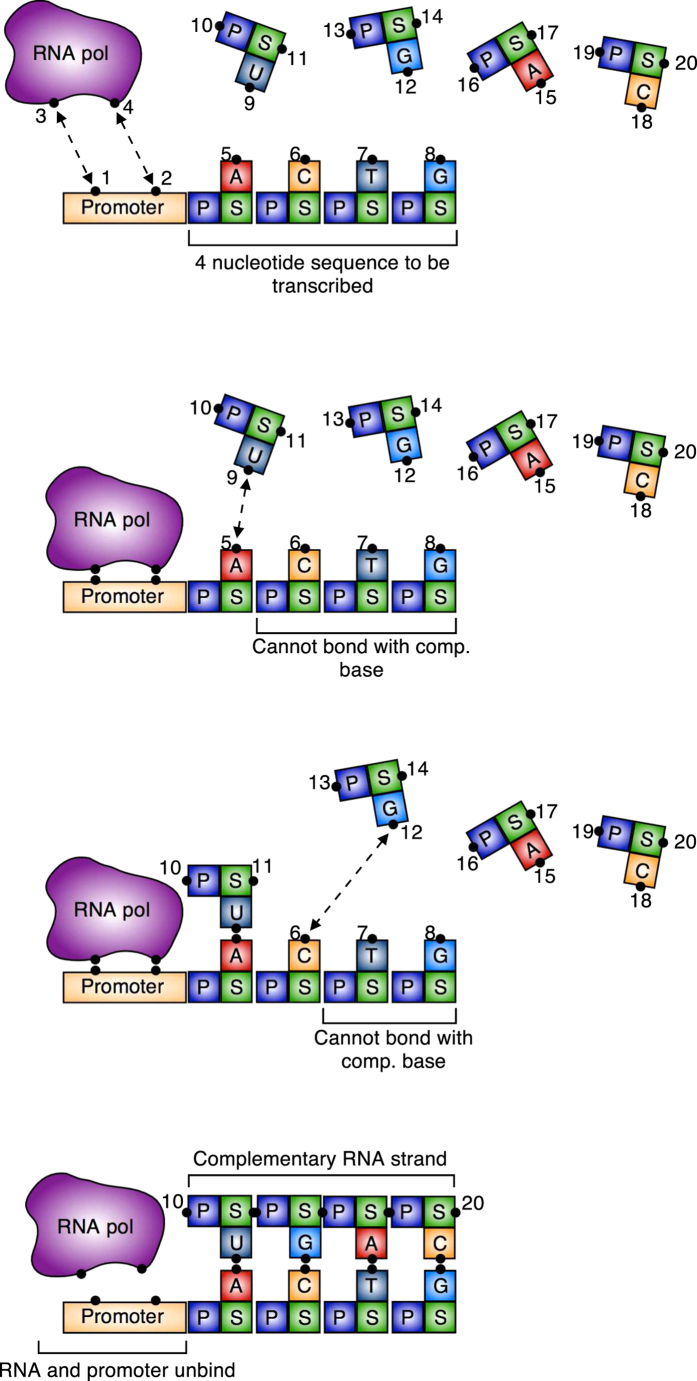
Simple DNA transcription model. Free base nucleotides cannot bind with the DNA strand until RNA pol binds with the promoter. When RNA pol is bound to the promoter, the free nucleotides bind to the DNA strand ACTG sequentially from left to right. Once the complementary strand has formed, the RNA pol unbinds from the promoter and then the complementary strand can diffuse away. Once the RNA pol has diffused far enough away, the bonds between the complementary base pairs turn off and the complementary strand can diffuse away. Dashed arrows between sites denotes an active potential. The P blocks denote a phosphate group and the S blocks denote a sugar group.

The final example is inspired by DNA transcription^27^,^28^. The model consists of a promoter region (sites 1 and 2) to which RNA polymerase (RNA pol) binds (sites 3 and 4), and a four nucleotide DNA strand, ACTG, to be transcribed (Fig. 10). As a first approximation of the transcription process, the movement of the RNA polymerase down the DNA chain and the unwinding/rewinding of the DNA have not been explicitly modeled.

In the absence of the RNA pol, the free nucleotides cannot bind to their complementary nucleotides in the 4 nucleotide DNA strand (ACTG = (5, 6, 7, 8)). Once RNA pol binds to the promoter, the first first nucleotide (A, atom 5) in the DNA strand can bind to the free version of its complementary nucleotide (U, atom 9). Before this binding happens, the remaining nucleotides in the strand (CTG, atoms 6, 7, 8) cannot bind with their (free) complementary nucleotides (atoms 12, 15, 18). Once A has bound to a free U nucleotide, the next nucleotide in the strand (C, atom 6) can bind with a free G nucleotide (atom 12), while the remaining two nucleotides (TG) still cannot bind with their complementary nucleotides. Once the free G has bound with C, the sugar and phosphate groups (atoms 11 and 13) on T and G can bind to start forming the backbone of the complementary DNA strand. This sequential process continues until each nucleotide in the original DNA strand ACTG has bound with its complementary nucleotide, resulting in the complementary RNA strand UGAC. At this point, the complementary strand and the RNA pol unbind from the original strand and promoter region, respectively.

Supplementary Table III lists the reaction potentials for each of the interacting pairs. The nucleotide base pairs interact via a hydrogen bond *ϕ*_H_, whereas the sugar and phosphate groups covalently bond through *ϕ*_SP_. The interaction potentials for the system can be easily read from this table (see Supplementary Information Sec. IV.A).

Let us step through the logic in the order the reaction occurs:

1. The bonds between the RNA pol and the promoter region (Φ_(1,3)_ and Φ_(2,4)_) are “on” except when the complementary chain has formed and is still attached to the original base strand.
2. The A-U bond (Φ_(5,9)_) is “on” when the RNA pol has bonded with the promoter and the complementary chain’s backbone has not fully formed. This second condition prevents the complementary strand from reattaching to the original DNA strand once it has been formed. It is “off” otherwise.
3. The C-G bond (Φ_(6,12)_) is “on” when all of following conditions are true: (1) RNA pol has bonded with the promotor, (2) the A-U bond has formed, and (3) the complementary backbone has not formed. It is “off” otherwise.
4. The sugar-phosphate group bond Φ_(11,13)_ turns “on” when (1) RNA pol has bonded with the promoter and (2) both the A-U and C-G bonds have formed. It remains “on” once the complementary backbone has formed. It is “off” otherwise.
5. The T-A bond (Φ_(7,15)_) is “on” when all of the following conditions are true: (1) RNA pol has bonded with the promotor, (2) the A-U and C-G bonds have formed, (3) the (11,13) sugar-phosphate bond has formed, and (4) the complementary backbone has not formed. It is “off” otherwise.
6. The sugar-phosphate group bond Φ_(14,16)_ turns “on” when (1) RNA pol has bonded with the promoter, (2) the A-U, C-G, and T-A bonds have formed, and (3) the (11, 13) sugar-phosphate bond has formed. It remains “on” once the complementary backbone has formed. It is “off” otherwise.
7. The G-C bond (Φ_(8,18)_) is “on” when all of the following conditions are true: (1) the conditions in (10) are true, (2) the T-A bond has formed, and (3) the (14, 16) sugar phosphate bond has formed. It is “off” otherwise.
8. The sugar-phosphate group bond Φ_(17,19)_ turns “on” when (1) RNA pol has bonded with the promoter, (2) the A-U, C-G, T-A, and G-C bonds have formed, and (3) the (11, 13) and (14, 16) sugar-phosphate bonds have formed. It remains “on” once the complementary backbone has formed. It is “off” otherwise.

A global potential derived from the above logic rules is given in Eq. (16). The exact form of the logic functions 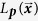 and the associated smooth encoding functions 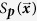 comprising the potential are given in the Supplementary Information. The derivation of the potential is not difficult, but lengthy. We refer the reader the Supplementary Information Sec. 4 for the details.


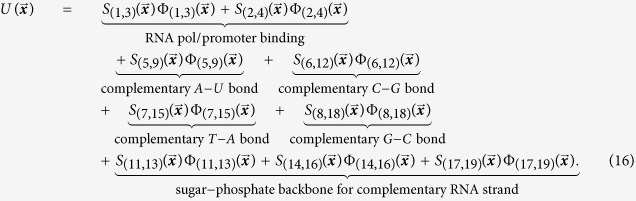


**Figure 11:**
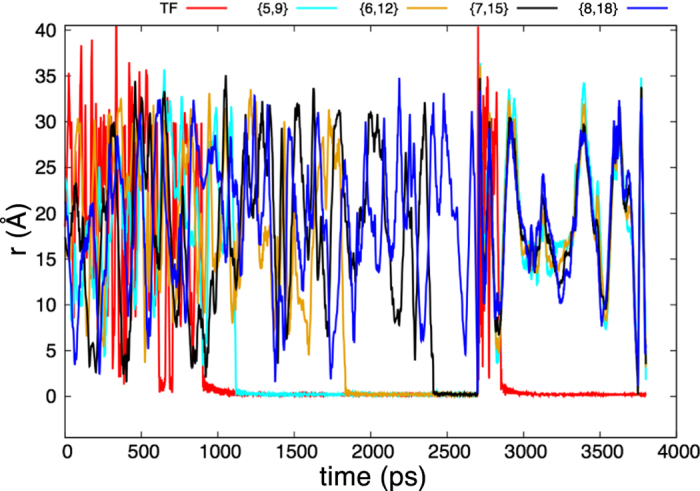
DNA transcription. The red trace (TF) corresponds to the distance between the RNA pol and the promoter region. *r*_5,9_ (cyan), *r*_6,12_ (gold), *r*_7,15_ (black), and *r*_8,18_ (blue) correspond to the sites on the complementary A-U, C-G, T-A, and G-C pairs from the base strand and the free nucleotides. At the start, the RNA pol and the free nucleotides diffuse around in space. Around 900 ps, the RNA pol binds to the promoter region (TF trace ≈ 0). The free nucleotides then bind in the the order of the designed logic. U binds to A (*r*_5,9_ ≈ 0) around 1100 ps; G binds with C (*r*_6,12_ ≈ 0) between 1800 and 1900 ps; A binds to T (*r*_7,15_ ≈ 0) around 2400 ps; and finally C binds to G (*r*_8,18_ ≈ 0) around 2700 ps. Once this final free nucleotide has bounded with its complement, the complementary chain is finished formed and unbinds as does the RNA pol. The RNA pol can rebind to the promoter region, but the complementary strand cannot rebind to the original DNA strand.

Figure 11 shows a trace of the pairwise distances between atoms for a typical simulation using this potential in LAMMPS (parameters in Supplementary Information Table IV). We use the same qualitative approximation of the force as was used in the inhibitor molecule example. For simplicity, all the potentials are taken to be Morse potentials. The red trace (TF) corresponds to the distance between the RNA pol and the promoter region. The variables *r*_5,9_ (cyan), *r*_6,12_ (gold), *r*_7,15_ (black), and *r*_8,18_ (blue) correspond to the sites on the complementary A-U, C-G, T-A, and G-C pairs from the base strand and the free nucleotides. At the start, the RNA pol and the free nucleotides diffuse around in space. Around 900 ps, the RNA pol binds to the promoter region (TF trace ≈0). The free nucleotides then bind in the the order of the designed logic. U binds to A (*r*_5,9_ ≈ 0) around 1100 ps; G binds with C (*r*_6,12_ ≈ 0) between 1800 and 1900 ps; A binds to T (*r*_7,15_ ≈ 0) around 2400 ps; and finally C binds to G (*r*_8,18_ ≈ 0) around 2700 ps. Once this final free nucleotide has bounded with its complement, the complementary chain has finished forming and unbinds as does the RNA pol. The RNA pol can rebind to the promoter region, but the complementary RNA strand cannot rebind to the original DNA strand. This is exactly the behavior designed into the potential. Supplementary Movie 3 in the Supplementary Information shows one simulation of the DNA transcription.

## Conclusions

We have developed and demonstrated a methodology and mathematical framework for obtaining an approximate interaction potential for a system which respects known coarse-level behavior. This methodology develops a semi-empirical model for the system by encoding the known coarse-level physics into logic functions that then modify simple pairwise potentials. Each logic function’s only role is to turn its associated pairwise potential on or off. A smooth multi-body interaction potential is obtained by replacing each logic function with a smoothed variant. The reader may wish to think of the resulting approximate potential as a linear combination of pairwise potentials where instead of the coefficients taking scalar values, they are encoding functions capturing the coarse-level logic.

Three relatively simple examples demonstrated our methodology: a simple inhibitor molecule mechanism, a chemical reaction with bond breaking, and a model inspired by DNA transcription. While these examples were simple and inspired by biophysical and chemistry applications, we stress that the methodology is quite general and not restricted to these application domains or only simple problems. Any system that is driven by a potential can utilize this methodology to its benefit.

The result of our procedure is the approximation of a complicated, high-dimensional potential with a lower-dimensional representation that still respects the relevant physics. A significant reduction in the dimensionality of the system is possible; instead of accounting for every interaction between a large number of components, we now only need as many variables as are needed to correctly model the coarse-level logic. In the bond breaking example, the potential capturing the logic was 8-dimensional, whereas the dimension of the configurations space was 12. The same system modeled at the quantum level is much more complicated. Since the bond breaking event is the relevant physics, the reduced order model is accurate enough for this purpose.

With this dimensional reduction, the ability to accurately simulate large, complicated systems within a computational design framework is feasible. The resultant models can be wrapped in an optimization loop as part of exploratory computational experiments, such as for the development of new drug therapies, or as part of an engineering design loop. This in turn allows for the faster and cheaper development of new technologies and products.

We note that the developed framework can be potentially used in reverse: not for approximation to a given physical process with coarse-grained logic given, but for design of molecular processes with logic prescribed by a designer. This is achieved by providing to the designer the specifications of molecules that can carry the logic out.

## Additional Information

**How to cite this article**: Thakur, G. S. *et al*. Programmable Potentials: Approximate N-body potentials from coarse-level logic. *Sci. Rep.* **6**, 33415; doi: 10.1038/srep33415 (2016).

## Supplementary Material

Supplementary Information

Supplementary Movie 1

Supplementary Movie 2

Supplementary Movie 3

## Figures and Tables

**Figure 1:**
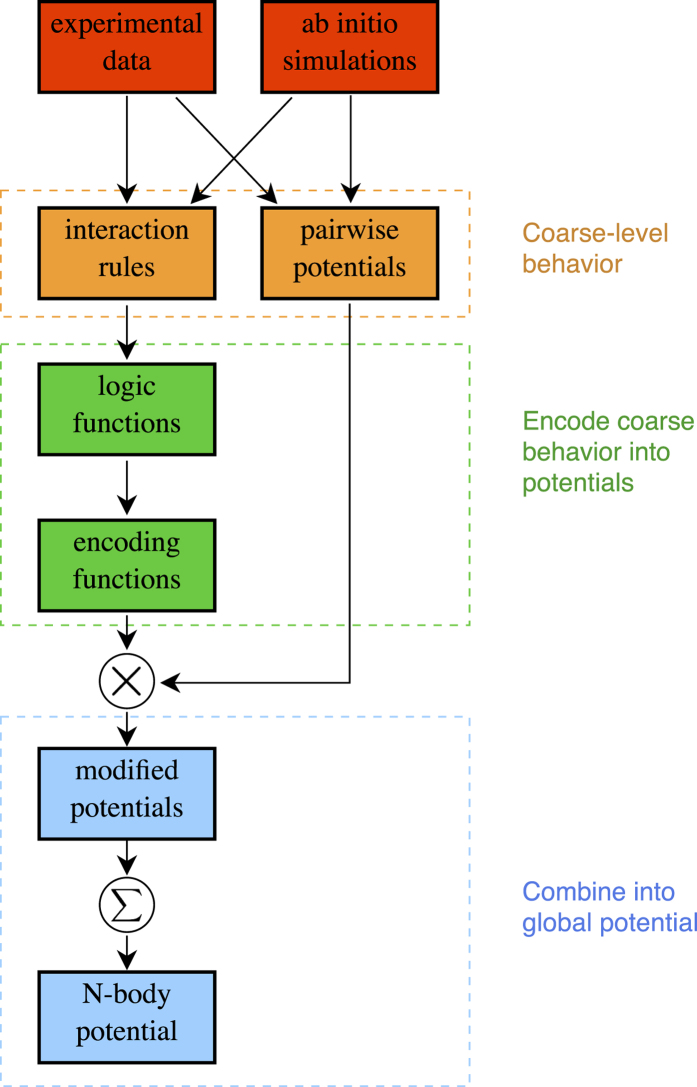
Flow chart of procedure. Using experimentally observed data and quantum calculations (red), we extract coarse-grain behavior (orange) namely: interactions rules and pairwise interaction potentials. This information is used to obtain an N-body potential (blue) for the system by employing the proposed formalism (green).

**Table 1:** Bond logic for the inhibitor molecule mechanism.

2 and 5 “close”	3 and 6 “close”	*L*_(2,3)_	*L*_(3,6)_	*L*_(2,5)_
0	0	1	1	1
0	1	0	1	0
1	0	0	0	1
1	1	0	0	0

**Table 2:** Potentials in chemical reaction model.

Potentials	Potential type	Equil. dist.	Interaction range
Φ_(1,3),1_	**AB** stable mol. bond	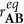	—
Φ_(1,3),2_	**AB** electron repulsion term	—	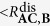
Φ_(2,5),1_	**AC** stable mol. bond	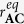	—
Φ_(2,5),2_	**AC** electron repulsion term	—	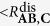

**Table 3:** Bond-breaking logic rules.

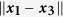	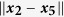	Φ_(1,3),1_	Φ_(1,3),2_	Φ_(2,5),1_	Φ_(2,5),2_
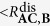	—	—	—	OFF	ON
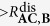	—	—	—	ON	OFF
—	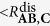	OFF	ON	—	—
—	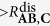	ON	OFF	—	—
