# Supplementary material for: Programmable Potentials: Approximate N-body potentials from coarse-level logic
Source: Sci Rep. 2016 Sep 27;6:33415. doi: 10.1038/srep33415 (PMC5037383; doi:10.1038/srep33415)
Supplement: Supplementary Information [file srep33415-s1.pdf]

Programmable Potentials: Approximate N-body potentials from coarse-level logic

Gunjan S. Thakur, Ryan Mohr, Igor Mezić

Programmable potentials pseudo-code

1. Identify all pairs of interacting atoms in the system, $\mathscr{I} = \{(i,j) : 1 \le i,j \le N, \text{atoms } i \text{ and } j \text{ interact}\}$.
2. For each $\boldsymbol{p} = (p_1, p_2) \in \mathscr{I}$, identify the multiplicity function $\mathfrak{m}(\boldsymbol{p})$, the number of distinct potential functions that capture different mechanisms through which atoms p_1 and p_2 interact.
3. Choose approximate/appropriate interaction potential model(s) $\Phi_{\boldsymbol{p},j}$, where $j \in \{1, \dots, \mathfrak{m}(\boldsymbol{p})\}$.
4. For each $\boldsymbol{p} \in \mathscr{I}$ and $j = 1, \dots, \mathfrak{m}(\boldsymbol{p})$, define the encoding function $S_{\boldsymbol{p},j}$:
   (a) Using experimental observations/prior knowledge/ab initio calculations, identify all atom pairs $\boldsymbol{q} \in \mathscr{I}$ affecting atom pair $\boldsymbol{p}$. Denote this set as
   $$A_{\boldsymbol{p},j} = \{\boldsymbol{q} \in \mathscr{I} : \boldsymbol{q} \text{ modifies the interaction between atom pair } \boldsymbol{p} \text{ for the mechanism } j\}.$$
   (b) Construct a logic table using the proximity of the atoms in each atom pair $\boldsymbol{q} \in A_{\boldsymbol{p},j}$ as inputs.
   (c) For each $\boldsymbol{q} \in A_{\boldsymbol{p},j}$, choose a value of $R(\boldsymbol{q}) > 0$ for $\ell_{\boldsymbol{q},R(\boldsymbol{q})}$ to define the proximity between the atoms in atom pair $\boldsymbol{q}$.
   (d) Form the logic function $L_{\boldsymbol{p},j}$ by using $\wedge, \vee, \neg$ to combine elements of the set of elementary logic functions $\{\ell_{\boldsymbol{q},R} : \boldsymbol{q} \in A_{\boldsymbol{p},j}\}$ according to the logic tables from 4(b).
   (e) Convert the logic function $L_{\boldsymbol{p},j}$ into a smooth "encoding function" $S_{\boldsymbol{p},j}$ using h-function (eq. (1)) replacement of each $\ell_{\boldsymbol{q},R(\boldsymbol{q})}$, $\boldsymbol{q} \in A_{\boldsymbol{p},j}$.
5. Compute equations (3) and (2) in the main text

Relevant equations

We record the functional forms of the h-functions that smooth the logic functions

$$h_{\alpha,n}(r) = \frac{1}{1 + (r/\alpha)^{2n}}, \qquad (0 \le \alpha \le \infty, n \in \mathbb{N}), \tag{1}$$

where $h_{0,n}(r)$ is defined to be identically 0 and $h_{\infty,n}(r)$ is defined to be identical 1. Additionally, we use the Morse functions in simulations.

$$\phi_{\text{Morse}}(r) = D\left(e^{-2a(r-r^{eq})} - 2e^{-a(r-r^{eq})}\right), \tag{2}$$

The variable D is the dissociation energy, r^{eq} is the equilibrium distance of the bond, and a is a parameter.

1 Simple inhibitor molecule mechanism

1.1 Inhibitor molecule simulations

* the cutoff value is a parameter LAMMPS uses to define the support of a potential. Particles outside the cutoff distance do not feel the effect of the potential.

Table 1: Inhibitor molecule simulation parameters.

Morse Potential, (2)	-
D	20 kcal/mol
a	2.2361
r^{eq}	2 Å
cutoff*	10 Å
Smoothing function, $h_{\alpha,n}$, (1)	-
α	$\frac{1}{3}$
n	5
LAMMPS (NVE) parameters	-
temp.	300 K
time step	0.01×10^{-15} sec
total sim. steps	5×10^{6}
comp. domain	26Å × 26Å
particle mass	2 grams/mol

1.2 Supplementary Video 1

This is a LAMMPS simulation of the inhibitor molecule example, driven by the potential given by equation (11) in the main manuscript. The simulation parameters are given by table 1.

The two blue atoms represent the receptor molecule **A**. The two green atoms represent the active molecule **B**. The two red atoms represent the inhibitor molecule **C**. The simulation starts with the active molecule **B** (green) bonded with the receptor molecule **A** (blue), with the inhibitor molecule **C** (red) away from the bonded pair. At approximately 00:02, the inhibitor molecule **C** (red) binds with the active molecule **B** (green), and the active molecule unbinds with the receptor molecule **A** (blue). The molecules remain in this state, diffusing around, until approximately 00:25, when the inhibitor molecule (red) unbinds with the active molecule (green) and binds with the receptor molecule (blue). At no time can the active molecule (green) rebind with the receptor molecule (blue) when in the presence of the inhibitor molecule (red).

2 Modeling a bond breaking chemical reaction

2.1 Energy transfer

Here, it is shown that the use of the smooth encoding function in the potential (as opposed to the logic function) allows the transfer of energy from **C** to **AB** so that, in order to break the bond, **C** must transfer energy equivalent to the bond dissociation energy $D_{\mathbf{AB}}$ of the **AB** bond $\Phi_{(1,3),1}$. Consider the event $\mathbf{AB}+\mathbf{C}$. Choose parameters for the h-functions so that the encoding functions satisfy

$$S_{(1,3),1}(\vec{\boldsymbol{x}}) \approx 0 \qquad \text{when } \|\boldsymbol{x}_2 - \boldsymbol{x}_5\| \lessapprox r^{eq}_{\mathbf{AC}}$$

and

$$S_{(2,5),1}(\vec{\boldsymbol{x}}) \approx 0 \qquad \text{when } \|\boldsymbol{x}_1 - \boldsymbol{x}_3\| \lessapprox r^{eq}_{\mathbf{AB}}.$$

See Fig. 1 for $S_{(1,3),1}(\vec{\boldsymbol{x}})$. The potential for this system is

$$U(\vec{\boldsymbol{x}}) = S_{(1,3),1}(\|\boldsymbol{x}_2 - \boldsymbol{x}_5\|)\Phi_{(1,3),1}(\|\boldsymbol{x}_1 - \boldsymbol{x}_3\|) + S_{(2,5),1}(\|\boldsymbol{x}_1 - \boldsymbol{x}_3\|)\Phi_{(2,5),1}(\|\boldsymbol{x}_2 - \boldsymbol{x}_5\|). \tag{3}$$

We will compute the energy transfer to **A** as **C** approaches **A**, when **A** and **B** are held fixed. Fix the position of $\boldsymbol{x}_1$ and $\boldsymbol{x}_3$ such that $\|\boldsymbol{x}_1 - \boldsymbol{x}_3\| = r^{eq}_{\mathbf{AB}}$ (**AB** bonded) and let $S_{(1,3),1}$ and $S_{(2,5),1}$ be the smoothed versions of the logic functions. The force acting on $\boldsymbol{x}_5$ due to $\boldsymbol{x}_2$ is

$$\begin{aligned} \boldsymbol{F}_{\mathbf{C}}(\vec{\boldsymbol{x}}) &= -\nabla_{\boldsymbol{x}_5} U(\vec{\boldsymbol{x}}) \\ &= -\nabla_{\boldsymbol{x}_5} S_{(1,3),1}(\|\boldsymbol{x}_2 - \boldsymbol{x}_5\|)\Phi_{(1,3),1}(\|\boldsymbol{x}_1 - \boldsymbol{x}_3\|) - S_{(2,5),1}(\|\boldsymbol{x}_1 - \boldsymbol{x}_3\|)\nabla_{\boldsymbol{x}_5}\Phi_{(2,5),1}(\|\boldsymbol{x}_2 - \boldsymbol{x}_5\|). \end{aligned}$$

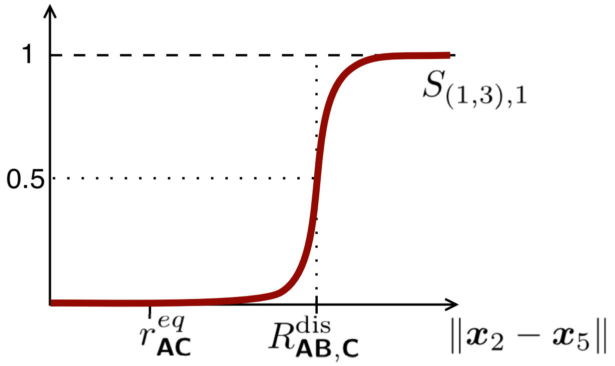

Figure 1: $S_{(1,3),1}$ encoding function turning the **AB** bond on and off.

For $\|\boldsymbol{x}_1 - \boldsymbol{x}_3\| = r^{eq}_{\mathsf{AB}}$, we have that $-\Phi_{(1,3),1}(r^{eq}_{\mathsf{AB}}) = D_{\mathsf{AB}}$. Furthermore, the assumptions above give that $S_{(2,5),1}(r^{eq}_{\mathsf{AB}}) \approx 0$. The force on $\boldsymbol{x}_5$ is approximately

$$\boldsymbol{F}_{\mathsf{C}}(\vec{\boldsymbol{x}}) \approx D_{\mathsf{AB}} \left(\frac{dS_{(1,3),1}(\|\boldsymbol{x}_2 - \boldsymbol{x}_5\|)}{dr} \right) \frac{\boldsymbol{x}_5 - \boldsymbol{x}_2}{\|\boldsymbol{x}_2 - \boldsymbol{x}_5\|}. \tag{4}$$

Using this force, we can compute the energy expended by **C** as **C** approaches **A** and hence the energy transfer from **C** to **A**. Assume **C** approaches **A** along the path $\boldsymbol{\ell}(r) = r\frac{\boldsymbol{x}_5 - \boldsymbol{x}_2}{\|\boldsymbol{x}_2 - \boldsymbol{x}_5\|}$, where r decreases from $+\infty$ to r^{eq}_{AC}:

$$\begin{aligned} E_{\mathsf{C}\to\mathsf{A}} &= -\int_{\boldsymbol{\ell}} \boldsymbol{F}_{\mathsf{C}}(\vec{\boldsymbol{x}}) \cdot d\boldsymbol{\ell} \\ &= -\int_{+\infty}^{r^{eq}_{\mathsf{AC}}} D_{\mathsf{AB}} \left(\frac{dS_{(1,3),1}(r)}{dr} \right) dr \\ &= D_{\mathsf{AB}} \left[S_{(1,3),1}(+\infty) - S_{(1,3),1}(r^{eq}_{\mathsf{AC}}) \right] \\ &\approx D_{\mathsf{AB}}, \end{aligned}$$

where we have used that $S_{(1,3),1}(+\infty) = 1$ and $S_{(1,3),1}(r^{eq}_{\mathsf{AC}}) \approx 0$.

It is clear that for appropriate choices of the parameters in the h-smoothing functions (eq. (1)) that this approximation can be made arbitrarily precise. Thus, the bond dissociation energy can be accurately accounted for using the smooth encoding functions that were used to model the bond breaking mechanism. The above argument is completely symmetric for the case when **AC** are bonded and **B** is approaching **A** from a distance greater than $R^{\text{dis}}_{\mathsf{AC},\mathsf{B}}$.

2.2 Bond breaking simulations

In the first simulation, the chemical reaction is unbiased. In the second, the reaction is biased toward a stable **AC** bond by increasing the depth **AC** potential well (2:1 well depth ratio). Simulation parameters are given in Table 2.

2.3 Supplementary Video 2

This is a simulation of the unbiased chemical reaction in LAMMPS using the parameters in table 2. Initially molecule **A** (pink/teal) and molecule **B** (purple/pink) are bonded and molecule **C** (teal/green) is free. The molecules remain in these states for awhile. During the simulation, molecule **C** approaches the bound **AB** pair, however, a reaction does not occur since **C** did not have enough energy to initiate a bond breaking. At around the 00:25 mark, a successful reaction occurs and molecule **A** (pink/teal) and **C** (teal/green) bound with molecule **B** becoming free. At approximately the 01:01 mark, the reverse reaction occurs, and **AB** reforms with **C** being free.

Table 2: Chemical reaction simulation parameters.

Parameter	Simulation 1 (unbiased)	Simulation 2 (biased)
h-function, (1)		
$n_{\mathsf{AB}} = n_{\mathsf{AC}}$	5	5
$R^{\text{dis}}_{\mathsf{AB},\mathsf{C}} = R^{\text{dis}}_{\mathsf{AC},\mathsf{B}}$	3 Å	3 Å
$\alpha_{\mathsf{AB}} = \alpha_{\mathsf{AC}}$	1/3Å	1/3Å
Morse potential, (2)		
$r^{eq}_{\mathsf{AB}} = r^{eq}_{\mathsf{AC}}$	2Å	2Å
$k_{\mathsf{AB}} = k_{\mathsf{AC}}$	200 kcal/(mol·Å)	200 kcal/(mol·Å)
D_{AB}	20 kcal/mol	10 kcal/mol
D_{AC}	20 kcal/mol	20 kcal/mol
$a_{\mathsf{AB}} = \sqrt{k_{\mathsf{AB}}/(2 \cdot D_{\mathsf{AB}})}$	$\sqrt{5}$	$\sqrt{10}$
$a_{\mathsf{AC}} = \sqrt{k_{\mathsf{AC}}/(2 \cdot D_{\mathsf{AC}})}$	$\sqrt{5}$	$\sqrt{5}$
LAMMPS parameters		
temp.	1000 K	1000 K
time step	0.01×10^{-15} sec	0.01×10^{-15} sec
total sim. steps	10^8	10^8
comp. domain	26Å × 26Å	26Å × 26Å
particle mass	2 grams/mole	2 grams/mol

3 DNA transcription model

3.1 DNA model reaction potentials

Table 3 lists the reaction potentials for each of the interacting pairs. The nucleotide base pairs interact via a hydrogen bond ϕ_{H}, whereas the sugar and phosphate groups covalently bond through ϕ_{SP}. The interaction potentials for the system can be easily read from this table. For example, active site 1 interacts with site 3 through a hydrogen bond ϕ_{H}. The corresponding potential is

$$\Phi_{(1,3)}(\vec{\boldsymbol{x}}) = \phi_{\mathsf{H}}(\|\boldsymbol{x}_1 - \boldsymbol{x}_3\|). \tag{5}$$

Active site 11 interacts with 13 via a sugar-phosphate group bond ϕ_{SP}:

$$\Phi_{(11,13)}(\vec{\boldsymbol{x}}) = \phi_{\mathsf{SP}}(\|\boldsymbol{x}_{11} - \boldsymbol{x}_{13}\|). \tag{6}$$

All of the other bonds are formed similarly from reading the table. We do not include the other sugar-phosphate group potentials (e.g., between 11 and 16). If these were included, we would also have to include logic functions that would prevent them from forming bonds before transcription begins. If we did not, then the free nucleotides could form a chain in free space which would prevent the proper transcription of the original DNA chain.

3.2 Logic functions for DNA transcription model

The logic functions for the base pairs, which are 1 when the complementary base pairs are bonded and 0 otherwise, are given in equations (7) - (10). The function L_{AU} determines when A and U are bonded or not, with obvious modifications for the other base pairs. The constant R^{eq}_{H} is the equilibrium length of the hydrogen bonds and $R_{\mathsf{H}} \geq R^{eq}_{\mathsf{H}}$ is used to determine whether the base pairs have bonded.

$$L_{\mathsf{AU}}(\vec{\boldsymbol{x}}) = \chi_{[0,R_{\mathsf{H}})}(\|\boldsymbol{x}_5 - \boldsymbol{x}_9\|), \tag{7}$$
$$L_{\mathsf{CG}}(\vec{\boldsymbol{x}}) = \chi_{[0,R_{\mathsf{H}})}(\|\boldsymbol{x}_6 - \boldsymbol{x}_{12}\|), \tag{8}$$
$$L_{\mathsf{TA}}(\vec{\boldsymbol{x}}) = \chi_{[0,R_{\mathsf{H}})}(\|\boldsymbol{x}_7 - \boldsymbol{x}_{15}\|), \tag{9}$$
$$L_{\mathsf{GC}}(\vec{\boldsymbol{x}}) = \chi_{[0,R_{\mathsf{H}})}(\|\boldsymbol{x}_8 - \boldsymbol{x}_{18}\|). \tag{10}$$

Table 3: Reaction potentials for DNA transcription. ϕ_{H} is the hydrogen bond potential between base pairs. ϕ_{SP} is the bond potential between the phosphate and sugar groups. **Cyan** numbers correspond to the RNA pol/promoter active sites. **Red** numbers correspond to active sites for base nucleotides, and **black** numbers correspond to sugar and phosphate groups. Each interaction potential for the system is listed twice in this table.

Active site, i	Interacts with, $j(i)$	Bond type
1	3	ϕ_{H}
2	4	ϕ_{H}
3	1	ϕ_{H}
4	2	ϕ_{H}
5	9	ϕ_{H}
6	12	ϕ_{H}
7	15	ϕ_{H}
8	18	ϕ_{H}
9	5	ϕ_{H}
10	–	–
11	13	ϕ_{SP}
12	6	ϕ_{H}
13	11	ϕ_{SP}
14	16	ϕ_{SP}
15	7	ϕ_{H}
16	14	ϕ_{SP}
17	19	ϕ_{SP}
18	8	ϕ_{H}
19	17	ϕ_{SP}
20	–	–

Similarly, we can define logic functions for the sugar-phosphate group bonds which form the backbone of the complementary chain.

$$L_{\mathsf{UG,SP}}(\vec{\boldsymbol{x}}) = \chi_{[0,R_{\mathsf{SP}})}(\|\boldsymbol{x}_{11} - \boldsymbol{x}_{13}\|), \tag{11}$$
$$L_{\mathsf{GA,SP}}(\vec{\boldsymbol{x}}) = \chi_{[0,R_{\mathsf{SP}})}(\|\boldsymbol{x}_{14} - \boldsymbol{x}_{16}\|), \tag{12}$$
$$L_{\mathsf{AC,SP}}(\vec{\boldsymbol{x}}) = \chi_{[0,R_{\mathsf{SP}})}(\|\boldsymbol{x}_{17} - \boldsymbol{x}_{19}\|), \tag{13}$$
$$L_{Backbone}(\vec{\boldsymbol{x}}) = L_{\mathsf{UG,SP}}(\vec{\boldsymbol{x}}) \wedge L_{\mathsf{GA,SP}}(\vec{\boldsymbol{x}}) \wedge L_{\mathsf{AC,SP}}(\vec{\boldsymbol{x}}). \tag{14}$$

The binding of the polymerase to the promoter region depends on whether the complementary chain has formed. Equation (15) is the corresponding logic function determining whether the complementary chain UGAC has formed or not. It is 1 when the chain has formed and 0 otherwise. The constant $R_{\mathsf{SP}} \geq R_{\mathsf{SP}}^{eq}$ is used to determine the whether the sugar and phosphate groups have bonded.

$$\begin{aligned} L_{\mathsf{UGAC}}(\vec{\boldsymbol{x}}) = {} & L_{\mathsf{AU}}(\vec{\boldsymbol{x}}) \wedge L_{\mathsf{CG}}(\vec{\boldsymbol{x}}) \wedge L_{\mathsf{TA}}(\vec{\boldsymbol{x}}) \wedge L_{\mathsf{GC}}(\vec{\boldsymbol{x}}) \\ & \wedge L_{Backbone}(\vec{\boldsymbol{x}}). \end{aligned} \tag{15}$$

The AND gates in the first line determine whether all the base nucleotides have formed hydrogen bonds with their complementary bases. The second line corresponds determines whether the sugar-phosphate backbone of the complementary chain has formed. The logic functions corresponding to the RNA pol and promoter binding ($\Phi_{(1,3)}$ and $\Phi_{(2,4)}$) are given in (16):

$$L_{(1,3)}(\vec{\boldsymbol{x}}) = L_{(2,4)}(\vec{\boldsymbol{x}}) = \neg L_{\mathsf{UGAC}}(\vec{\boldsymbol{x}}). \tag{16}$$

The logic function

$$L_{\mathsf{RNA/Pr}}(\vec{\boldsymbol{x}}) = \chi_{[0,R_{\mathsf{RNA/Pr}})}(\|\boldsymbol{x}_1 - \boldsymbol{x}_3\|) \wedge \chi_{[0,R_{\mathsf{RNA/Pr}})}(\|\boldsymbol{x}_2 - \boldsymbol{x}_4\|) \tag{17}$$

determines whether the RNA polymerase has bonded to the promoter region.

The logic functions for the A-U, C-G, T-A, and G-C bonds are given in equations (18) - (21), respectively. As can be seen, each step of the process requires an additional AND gate with the logic function determining the bonding of the previous base pair.

$$L_{(5,9)}(\vec{\boldsymbol{x}}) = L_{\mathsf{RNA/Pr}}(\vec{\boldsymbol{x}}) \wedge (\neg L_{Backbone}(\vec{\boldsymbol{x}})) \tag{18}$$
$$L_{(6,12)}(\vec{\boldsymbol{x}}) = L_{\mathsf{RNA/Pr}}(\vec{\boldsymbol{x}}) \wedge L_{\mathsf{AU}}(\vec{\boldsymbol{x}}) \wedge (\neg L_{Backbone}(\vec{\boldsymbol{x}})) \tag{19}$$
$$L_{(7,15)}(\vec{\boldsymbol{x}}) = L_{\mathsf{RNA/Pr}}(\vec{\boldsymbol{x}}) \wedge L_{\mathsf{AU}}(\vec{\boldsymbol{x}}) \wedge L_{\mathsf{CG}}(\vec{\boldsymbol{x}}) \wedge L_{\mathsf{UG,SP}}(\vec{\boldsymbol{x}}) \wedge (\neg L_{Backbone}(\vec{\boldsymbol{x}})) \tag{20}$$
$$L_{(8,18)}(\vec{\boldsymbol{x}}) = L_{\mathsf{RNA/Pr}}(\vec{\boldsymbol{x}}) \wedge L_{\mathsf{AU}}(\vec{\boldsymbol{x}}) \wedge L_{\mathsf{CG}}(\vec{\boldsymbol{x}}) \wedge L_{\mathsf{UG,SP}}(\vec{\boldsymbol{x}}) \wedge L_{\mathsf{TA}}(\vec{\boldsymbol{x}}) \wedge L_{\mathsf{GA,SP}}(\vec{\boldsymbol{x}}) \wedge (\neg L_{Backbone}(\vec{\boldsymbol{x}}))\,. \tag{21}$$

In each of these equations, the logic term $(\neg L_{Backbone}(\vec{\boldsymbol{x}}))$ prevents the complementary chain from reattaching to the original chain once it has been fully formed.

The logic functions for the sugar-phosphate bonds are given in equations (22) - (24). They prevent the complementary chain from spontaneously forming before transcription begins, prevents transcription errors, and makes sure the complementary chain stays together once it detaches from the original chain. This last objective is achieved by the logic terms following the OR ($\vee$) operation.

$$L_{(11,13)}(\vec{\boldsymbol{x}}) = \big(L_{\mathsf{RNA/Pr}}(\vec{\boldsymbol{x}}) \wedge L_{\mathsf{AU}}(\vec{\boldsymbol{x}}) \wedge L_{\mathsf{CG}}(\vec{\boldsymbol{x}})\big) \vee (L_{\mathsf{GA,SP}}(\vec{\boldsymbol{x}}) \wedge L_{\mathsf{AC,SP}}(\vec{\boldsymbol{x}})) \tag{22}$$
$$L_{(14,16)}(\vec{\boldsymbol{x}}) = \big(L_{\mathsf{RNA/Pr}}(\vec{\boldsymbol{x}}) \wedge L_{\mathsf{AU}}(\vec{\boldsymbol{x}}) \wedge L_{\mathsf{CG}}(\vec{\boldsymbol{x}}) \wedge L_{\mathsf{TA}}(\vec{\boldsymbol{x}}) \wedge L_{\mathsf{UG,SP}}(\vec{\boldsymbol{x}})\big) \vee (L_{\mathsf{UG,SP}}(\vec{\boldsymbol{x}}) \wedge L_{\mathsf{AC,SP}}(\vec{\boldsymbol{x}})) \tag{23}$$
$$\begin{aligned} L_{(17,19)}(\vec{\boldsymbol{x}}) = {} & \big(L_{\mathsf{RNA/Pr}}(\vec{\boldsymbol{x}}) \wedge L_{\mathsf{AU}}(\vec{\boldsymbol{x}}) \wedge L_{\mathsf{CG}}(\vec{\boldsymbol{x}}) \wedge L_{\mathsf{TA}}(\vec{\boldsymbol{x}}) \wedge L_{\mathsf{GC}}(\vec{\boldsymbol{x}}) \wedge L_{\mathsf{UG,SP}}(\vec{\boldsymbol{x}}) \wedge L_{\mathsf{GA,SP}}(\vec{\boldsymbol{x}})\big) \\ & \vee (L_{\mathsf{GA,SP}}(\vec{\boldsymbol{x}}) \wedge L_{\mathsf{UG,SP}}(\vec{\boldsymbol{x}})) \end{aligned} \tag{24}$$

Using the normal h-function replacement of the indicator functions, a global potential driving the DNA

transcription process is

$$
\begin{aligned}
U(\vec{\boldsymbol{x}}) = & \underbrace{S_{(1,3)}(\vec{\boldsymbol{x}})\Phi_{(1,3)}(\vec{\boldsymbol{x}}) + S_{(2,4)}(\vec{\boldsymbol{x}})\Phi_{(2,4)}(\vec{\boldsymbol{x}})}_{\text{RNA pol/promoter binding}} \\
& + \underbrace{S_{(5,9)}(\vec{\boldsymbol{x}})\Phi_{(5,9)}(\vec{\boldsymbol{x}})}_{\text{complementary A-U bond}} + \underbrace{S_{(6,12)}(\vec{\boldsymbol{x}})\Phi_{(6,12)}(\vec{\boldsymbol{x}})}_{\text{complementary C-G bond}} + \underbrace{S_{(7,15)}(\vec{\boldsymbol{x}})\Phi_{(7,15)}(\vec{\boldsymbol{x}})}_{\text{complementary T-A bond}} + \underbrace{S_{(8,18)}(\vec{\boldsymbol{x}})\Phi_{(8,18)}(\vec{\boldsymbol{x}})}_{\text{complementary G-C bond}} \\
& + \underbrace{S_{(11,13)}(\vec{\boldsymbol{x}})\Phi_{(11,13)}(\vec{\boldsymbol{x}}) + S_{(14,16)}(\vec{\boldsymbol{x}})\Phi_{(14,16)}(\vec{\boldsymbol{x}}) + S_{(17,19)}(\vec{\boldsymbol{x}})\Phi_{(17,19)}(\vec{\boldsymbol{x}}).}_{\text{sugar-phosphate backbone for complementary RNA strand}} \qquad (25)
\end{aligned}
$$

3.3 DNA Simulation parameters

For simplicity, all of the potentials listed in Table 3 are chosen to be Morse potentials, (2).

Table 4: DNA transcription simulation parameters

units	non-dimensional
computational box ($L \times W$)	40×30
LAMMPS (NVE) parameters	
temp.	0.5
Δt	0.001
# times steps	3.8×10^6
Morse potential	all atoms
Dissociation energy, D	100
a	2.0
r_{eq}	0.2
h-function parameters	all atoms
n	3
R (all proximity functions)	1.5
$\alpha = 1/R$	$\frac{2}{3}$

3.4 Supplementary Video 3

This is a LAMMPS simulation of the DNA transcription process driven by potential (25) with the simulation parameters in table 4. In the simulation, the RNA promoter region is described by just a single site rather than two sites and similarly for the RNA polymerase. This does not effect the qualitative behavior of the simulation; the only effect is that the RNA pol/promoter binding term, $S_{(1,3)}(\vec{\boldsymbol{x}})\Phi_{(1,3)}(\vec{\boldsymbol{x}})+S_{(2,4)}(\vec{\boldsymbol{x}})\Phi_{(2,4)}(\vec{\boldsymbol{x}})$, in (25) becomes a single encoding function/potential pair.

In the video, the promoter region is given by an isolated (and fixed position) teal ball in the bottom left of the window. The **ACTG** DNA chain to be transcribed is positioned directly to the right of the promoter atom and is represented by a yellow-black-red-teal horizontal chain of atoms. This chain remains in a fixed position throughout the simulation. The complementary, free nucleotides are given by the molecules formed from three atoms (two teal, one other color). The RNA polymerase is given by the free, single teal atom that is situated vertically between the free nucleotides and the DNA chain.

In the simulation, the RNA pol binds to the promoter region at around the 00:08 mark. At approximately the 00:09 mark, the free **U** nucleotide (blue) attaches to the fixed **A** nucleotide (yellow) in the DNA chain. At approximately the 00:15 mark, the free **G** nucleotide (black) binds to **C** (black) in the DNA chain. The free **A** (red) binds to the **T** (red) in the DNA chain, at around the 00:20 mark. The final free nucleotide (**C**, pink) binds to the final unbounded nucleotide (**G**, teal) in the DNA chain at approximately the 00:22 mark. Immediately, the conditions for the unbinding of the complementary chain and the RNA polymerase are met and they are free to diffuse in away.
